# Interatrial Shunt Devices—A Potential Therapy for Mitral Stenosis Following MitraClip

**DOI:** 10.1016/j.jscai.2023.101033

**Published:** 2023-05-15

**Authors:** Evan Harmon, Anirudh Kumar, Serge Harb, Samir Kapadia, Grant Reed

**Affiliations:** Cleveland Clinic Foundation, Cleveland, Ohio

**Keywords:** atrial septal defect, iatrogenic mitral stenosis, interatrial shunt devices, MitraClip

**Figure 1:**
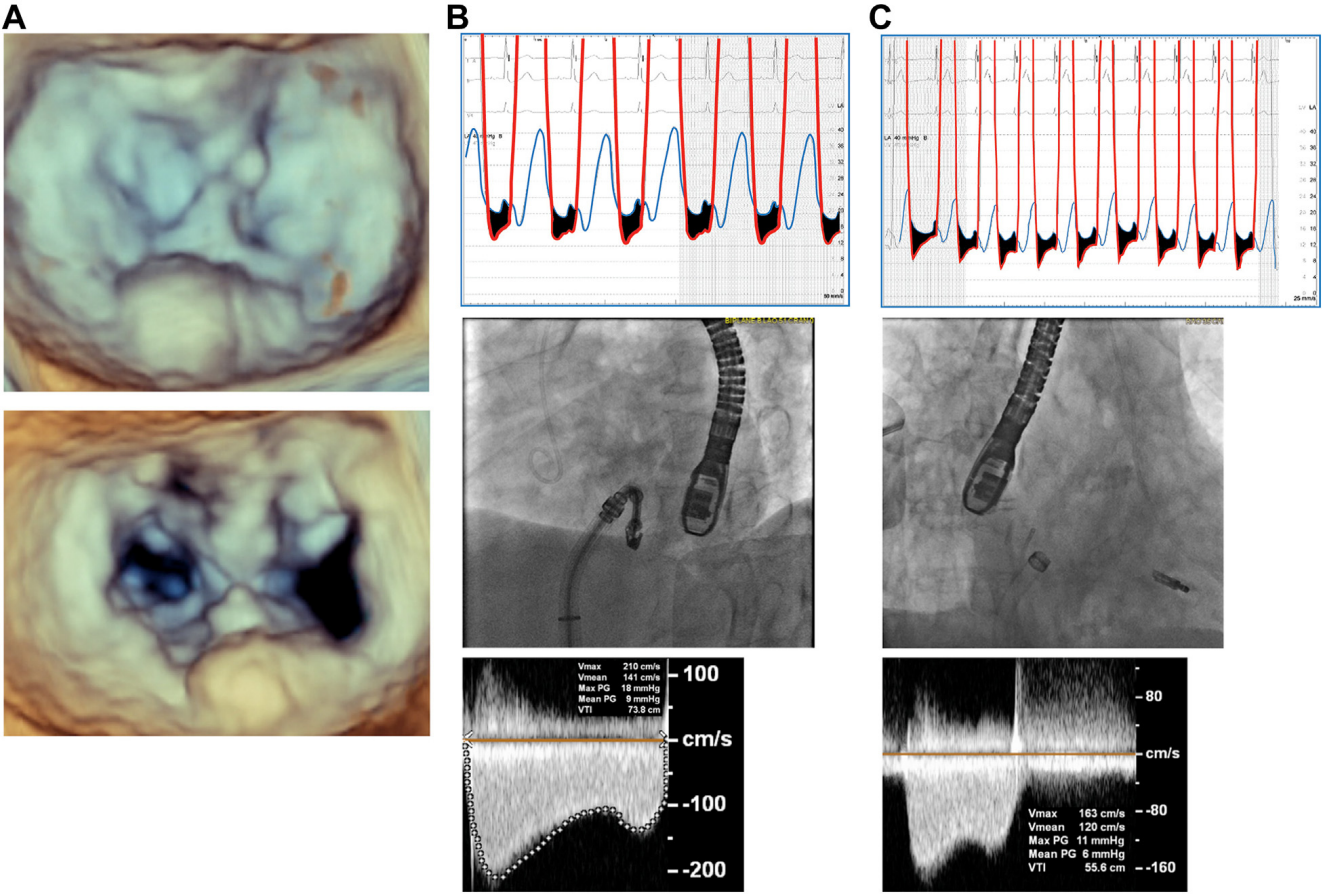
**Three-dimensional echocardiographic mitral valve reconstruction and hemodynamic data.** (**A**) Pre-MitraClip and post-MitraClip 3-dimensional echocardiographic reconstruction of the mitral valve, demonstrating severe posterior mitral annular calcification. (**B**) Hemodynamics after grasping both leaflets with the NT MitraClip device still attached to the guide. (**C**) Hemodynamics after retraction of the guide into the right atrium after deployment.

Iatrogenic mitral stenosis (MS) is an important factor when determining candidates for MitraClip, particularly when requiring multiple clips or in patients with preexisting MS. We present an 87-year-old woman with severe mitral regurgitation (MR). Pre-echocardiogram noted preserved biventricular function, moderate tricuspid regurgitation, and right ventricular systolic pressure of 47 mm Hg. MR mechanism was P2 prolapse with mean mitral gradient (MG) of 5 mm Hg and severe posterior mitral annular calcification. Invasive hemodynamics noted left atrial (LA) pressure of 20 mm Hg with V waves to 50 mm Hg and left ventricular end-diastolic pressure of 18 mm Hg. One NT MitraClip device was placed at the A2/P2 segments with trivial residual MR, but with MG of 9 mm Hg and mean LA pressure of 22 mm Hg (Figure 1). After removal of the MitraClip guide into the inferior vena cava, there was a left-to-right shunt across the interatrial septum with improved MG of 6 mm Hg, mean LA pressure of 12 mm Hg, V waves of 24 mm Hg, and pulmonary vein flow normalization. Follow-up transthoracic echocardiogram within 24 hours confirmed stable MG of 6 mm Hg. However, subsequent echocardiogram performed 2 months postprocedure revealed closure of the iatrogenic atrial septal defect with MG then at 13 mm Hg with a heart rate of 83 bpm. Six months postprocedure, the MG remained at 13 mm Hg (heart rate, 85 bpm).

The REDUCE-LAP HF II study assessed the effect of an 8-mm interatrial shunt device (ISD) on heart failure patients with ejection fraction of ≥40% and exercise-induced pulmonary capillary wedge pressure of ≥25 mm Hg without right heart failure.^1^ After a 2-year follow-up, there was no difference in heart failure hospitalization between ISD placement and sham control. We believe that where REDUCE-LAP HF II failed, the ongoing RELIEVE-HF trial (NCT03499236) will succeed, as RELIEVE-HF will randomize patients to a smaller 5.1-mm V-Wave ISD and will include patients with significant systolic dysfunction.

The therapeutic premise of ISDs in MS patients dates to the early 1900s, when Lutembacher^2^ described improved symptomatology among patients with MS and concomitant congenital secundum atrial septal defect (ASD). In this setting, the ASD functions as a “pop-off valve” for elevated LA pressures in patients with preserved right heart function. This case report demonstrates the acute hemodynamic benefit of creating an iatrogenic ASD in a patient with elevated MG after MitraClip, which then dissipated as the ASD closed over time. By creating a left-to-right shunt, the MG and LA pressure were both significantly lowered—which may be particularly beneficial at higher heart rates and dynamic conditions of left ventricular loading postprocedure. Although patients with MS will not be enrolled in RELIEVE-HF, they are often without treatment options and may benefit for similar reasons. Perhaps, the future of ISDs has come full circle, with potential application to patients with calcific MS without right heart failure or those who develop MS after MitraClip, as highlighted in this case. In addition to improving symptoms, this therapy may allow operators to better treat the MR with less concern of creating iatrogenic MS.^3^^,^^4^
